# Analysis of the Costs and Reach of Transportation Services to Reduce Barriers to Opioid Use Disorder (OUD) Treatment: Evidence from the Kentucky HEALing Communities Study

**DOI:** 10.21203/rs.3.rs-7999770/v1

**Published:** 2025-11-20

**Authors:** Drew D. Speer, Joshua L. Bush, Don Lochana Ekanayake, Julie Nakayima, Michael M. Goetz, Jennifer Miles, Elizabeth Larimore, Hannah K. Knudsen, Sharon L. Walsh, Kathryn E. McCollister

**Affiliations:** University of Kentucky; University of Kentucky; University of Miami Miller School of Medicine; University of Kentucky; University of Kentucky; University of Kentucky; University of Kentucky; University of Kentucky; University of Kentucky; University of Miami Miller School of Medicine

**Keywords:** transportation, substance use, economics, OUD policy, barriers to care

## Abstract

**Background:**

Medications for opioid use disorder (MOUD) have been proven to be the most effective method for treating individuals with opioid use disorder (OUD). Individuals who are retained on methadone and buprenorphine treatment have a decreased risk of mortality when compared to others with OUD. However, many individuals in need of MOUD face transportation barriers that prevent them from having the requisite access to treatment.

**Methods:**

As part of the evidence-based practices (EBPs) implemented throughout the HEALing (Helping to End Addiction Long-term^®^) Communities Study (HCS), strategies to reduce transportation barriers to receiving methadone and buprenorphine at partner organizations were identified and implemented. Multiple strategies were employed including transportation agency contracts, bus passes, rideshare services, leased agency vans, and fuel cards. This study provides a description of the number of unique riders, including demographic and geographic characteristics and the costs of providing these transportation services across participating counties.

**Results:**

Throughout 2023, we partnered with thirty agencies across eight counties to administer $437,481.69 in transportation support services to 7,923 individuals. These services were distributed primarily to individuals who were white (81.8%; 6,483), female (51.9%; 4,112) and between the ages of 35–54 (59.8%; 4,737). The number of individuals reached and expenses were largest among the five urban counties (5,999; $318,317.15), which averaged $53.06 per person. Rural counties averaged $61.94 per person reached (1,924; $119,164.54). A survey was administered among a subpopulation of these individuals, and among this group, 813 of 958 (84.9%) identified transportation as a major barrier to obtaining/staying in care with MOUD. Within this subgroup, 262 individuals (27.3%) made 377 unique transportation service requests (27.7% of all subgroup requests), which included vehicle repairs (81), peer recovery drivers for rides directly to MOUD appointments (73), and bus passes (70).

**Conclusions:**

By gaining an understanding of the population benefiting from these services and the costs required to address this barrier to care, we may more effectively inform decision-makers on the resources needed to link and retain individuals in treatment.

**Trial registration:**

ClinicalTrials.gov, NCT04111939. Registered 30 September 2019, https://clinicaltrials.gov/ct2/show/NCT04111939

## Introduction

The Centers for Disease Control and Prevention reported that drug overdose deaths involving opioids exceeded 80,000 for the third consecutive year in 2023 (1). Nearly 70% of these overdose deaths involved synthetic opioids, primarily fentanyl (1). To address drug overdose mortality, in 2021, the U.S. Department of Health & Human Services (DHHS) released an Overdose Prevention Strategy, which aims to support four strategic priorities: primary prevention, harm reduction, evidence-based treatment, and recovery support (2). Within the evidence-based treatment strategies, two FDA-approved medications for opioid use disorder (MOUD), buprenorphine and methadone, are effective in reducing opioid use and overdose among individuals with opioid use disorder (OUD) (3). However, uptake of these life-saving medications is low with estimates suggesting that only around 25% of individuals with OUD receive MOUD (4,5). Moreover, retention in MOUD care is poor and discontinuing treatment increases the risk of relapse and overdose (6).

Access to transportation is one of the most widely cited barriers to engaging in healthcare, and lack of transportation is a key barrier to MOUD retention (7–15). People with chronic diseases, such as OUD, are disproportionately impacted by transportation barriers (16–19). Individuals with OUD are often required to visit opioid treatment providers daily or near daily, or physician offices multiple times a week, especially in the first few weeks of recovery, to setup their treatment plans and calibrate medication dosages (20). These requirements, while important to prevent relapse, exacerbate the transportation burdens many individuals face as a result of limited vehicle access/reliability or required travel time through public transit (21,22). Transportation barriers are especially common among rural populations, where fewer than 40% of MOUD providers are within walking distance of any form of public transportation and rural residents may spend five times longer traveling to a medical appointment compared to urban residents (21,23).

Efforts to address transportation challenges for people with OUD are poorly documented and therefore not well understood. In this study, we explore this critical knowledge gap by evaluating the costs and reach of a transportation assistance program for individuals with OUD that was implemented as part of the HEALing (Helping to End Addiction Long-term^®^) Communities Study (HCS). HCS was a multi-site study in four states (KY, OH, NY and MA) focused on implementing evidence-based practices (EBPs) to significantly reduce opioid-related overdose fatalities in 67 urban and rural counties (24). As part of this study, to address barriers to the uptake of and retention in MOUD treatment, EBPs were implemented to improve delivery of OUD services across counties. The overall HCS KY barrier relief initiative targeted numerous community needs, including transportation, housing and rental assistance, cell phones, healthcare and legal services. Because lack of access to affordable and reliable transportation was one barrier frequently reported, HCS KY designed and implemented a Transportation Assistance Program (TAP) to improve transportation options within collaborating partner agencies in healthcare and behavioral health settings (19). This paper first describes the HCS KY TAP and then provides results of the cost analysis of this program and the number of individuals impacted through measures of program reach.

## Background

### Determination of Transportation Needs and Community Resources

The implementation of the HCS KY TAP within MOUD partner agencies was led by HCS KY implementation facilitators. Partner agencies primarily consisted of Opioid Treatment Providers or Office-Based Opioid Treatment facilities within each county. These individuals conducted small group interviews with each partner agency to determine the specific obstacles faced by the populations served, including challenges to access and retention in MOUD treatment programs. During these interviews, sixty-six of the seventy-six employees (86.8%) from thirty agencies interviewed identified transportation as a key barrier to patient retention. After the interview, the implementation facilitator summarized key barriers to retention and forwarded interview transcripts to HCS KY leadership. Based on responses to these interviews, HCS KY leadership determined which types of assistance would be offered to each agency. From these recommendations, thirty partner agencies were identified to receive transportation barrier relief services through the HCS KY TAP (25).

Following the implementation facilitator interviews with partner organizations, the HCS KY team explored various methods to improve client transportation access. Public transportation and Medicaid transportation services were surveyed earlier during a landscape analysis conducted prior to the HCS intervention implementation, which helped to establish contacts and standard operating procedures for accessing public transportation passes or scheduling ride services. The HCS KY team also identified churches, local taxi services, and third-party ride service transportation vendors as potential options for local agencies. The HCS KY team prioritized pre-paid public transportation passes, fuel, and Uber gift cards to partner organizations due to difficulties in the payment process when working with local taxi services and ride service vendors.

Based on the interview findings and available local resources (which differed considerably among communities), a tiered funding model was developed for partner agencies. This model considered the number of clients served by each organization as a primary factor in determining financial allocation, with larger patient volumes considered reflective of greater transportation needs. Organizations were eligible for up to $1,000 per month if fewer than 100 MOUD patients were seen, $2,000 per month for 101–200 patients, and so on. These tiers were used for agencies in both rural and urban locations.

The implementation facilitator arranged meetings to discuss these services with partner agencies. HCS KY team members discussed agency contractual responsibilities and provided workflow information for orders and replenishments. Once an agency accepted the offer of transportation assistance, HCS KY established contracts with those agencies and relevant third parties to provide the agreed upon mix of transportation options. Implementation facilitators worked with organizations to design internal client protocols and organizational support for working with the HCS KY team. The HCS KY TAP team managed the logistics of organizing the purchase, delivery, data collection, and troubleshooting of transportation programs.

### Transportation Program Design and Administration

The HCS KY TAP was composed of two mechanisms to provide funding for individuals in need. First, fuel cards, bus passes, rideshare reimbursement, and/or funds were issued to a partner agency to provide transportation directly to their clients (“partner agency model”). Second, recovery coaches from Voices of Hope – Lexington (VOH), trained individuals with lived experience, and care navigators from Bluegrass Care Navigators (BCN), certified social workers or nurses, who were placed at partner agencies through HCS KY, were able to request transportation-related funds to cover expenses that would support individuals in accessing or continuing treatment.

In the partner agency model, clinical staff working in partner organizations identified individuals experiencing transportation barriers and offered them support. Clients receiving MOUD retention services or initiating treatment (e.g., first appointment) at any of the partner agencies in the eight HCS KY counties were eligible to receive HCS KY TAP services. There were no client income limitations in place for transportation assistance requests. For those with access to a vehicle, support included fuel cards. For participants without a vehicle, solutions included public transportation passes (bus passes), transportation service financing (transportation service vendor invoicing and funding for vehicles leased by agency partners), and rides arranged by partner agencies (e.g., Uber gift cards).

For partner agencies, data collection and governance methods were employed to track transportation assistance and barrier relief efforts, including demographic breakdowns and service types provided. These data served two functions: collecting data on the reach of our transportation services and reporting to the university regarding the use and management of grant funds.

A Transportation Request Tracker was implemented using Smartsheet^™^ to collect data submitted by agencies for replenishments or reimbursement for transportation services provided to their clients and the cost of those services. This tracker collected data on the type of assistance being requested by the agency, quantity of cards/passes (if applicable), the request submission date, and whether it was approved by the HCS KY Team.

In the second model, recovery coaches and care navigators implementing retention-related services within MOUD partner agencies could access funding that addressed patients’ transportation needs as well as other social needs. Recovery coaches and care navigators were embedded in multiple MOUD partner agencies to provide patient recovery support and targeted assistance for those most in need of retention services. Transportation support included bus passes and fuel cards like the HCS KY TAP, but also included funds for acquiring driver’s licenses, car title expenses, purchasing non-motorized vehicles, and a new ride service program where clients could ride with recovery coaches to and from MOUD appointments, later referred to as direct delivery (26). To be eligible to receive assistance through the HCS KY TAP, clients had to be 18 years of age or older and receive services in one of the eight HCS KY counties or from an approved Opioid Treatment Provider in a nearby county.

The recovery coaches and care navigators implementing the transportation assistance program surveyed clients to collect additional information at the time of the transportation assistance request to identify barriers preventing or creating challenges for MOUD treatment program participation. Of the barriers identified, transportation was most commonly cited and was reported as a barrier for 813 of the 958 individuals completing the survey (84.9%). Recovery coach and care navigator partners fielded 1,362 requests for barrier relief services submitted by 958 individuals, of which 262 of the 958 (27.3%) individuals made 377 of the 1,362 (27.7%) requests for assistance to address transportation needs. Across all barriers among this sub-population, transportation requests were second only to housing-related needs. The most common transportation service sub-types were the need for vehicle repairs, bus passes, or a recovery coach driver to transport that individual directly to MOUD treatment.

## Methods – Cost and Reach Analysis

The cost analysis is framed from the community’s perspective, (i.e., a county), which represents multiple agencies supporting the TAP. The goal is to highlight the resources that would be needed in any community planning to incorporate transportation assistance services.

This study uses data from eight HCS KY counties (five urban, three rural). Data on services and expenditures were collected during the HCS EBP implementation period of January 2023 through December 2023 (12 months). The HCS KY team used REDCap tools hosted at the University of Kentucky to collect data on the individuals reached by the HCS KY TAP across the eight counties (25, 27, 28). Partner agencies submitted a form through the REDCap project for every instance where transportation assistance was provided to a client (i.e., a bus pass, fuel card, Uber/Lyft ride, taxi, Uber/Lyft gift card, or other). Data regarding the number of unique individuals served through the transportation assistance partnerships included a de-identified unique client ID, the type of assistance and the date it was provided, the client’s county of residence, the initials of the agency staff who completed the form, and client demographic information (age, race, ethnicity, and sex). In some cases, it was not feasible for the partner agency to report person-level data on the transportation assistance they were providing. For these cases, HCS KY received aggregate counts from the agency of the number of individuals served each month while the strategy was active. The aggregated data were then recorded in a separate, centralized REDCap Tracker, organized by partner agency, county, month, and strategy. Data points collected included a total count of individuals served and aggregated demographics (i.e., age, race/ethnicity, and sex).

To evaluate cost and reach, we analyzed the data collected from these agencies to understand populations served, transportation services provided, the method of payment or reimbursement, and new services adopted with funding support from the HCS. With few exceptions, HCS KY participating agencies that were focused on the strategy of retaining people on MOUD had to develop de novo transportation assistance service.

We define reach as the number of unique individuals receiving HCS KY TAP services through 30 partner agencies representing two sectors (behavioral health and healthcare). This measure is used to calculate “average cost per individual reached” as a cost summary statistic.

Transportation costs were captured in three categories: vouchers, ride share and other transportation services, and direct delivery provided by recovery coach partners. Cost data were organized using Smartsheet^™^ and exported and analyzed in Excel^®^ (Microsoft Corporation, 2024). Summary cost statistics report total cost per county, costs segmented by rural/urban counties, and total costs across all transportation services delivered. To preserve anonymity, counties are identified in this manuscript by their urban or rural status (U or R) and a number identifier.

Reach data were exported and analyzed using SAS v9.4 (Cary, NC). This study protocol (Pro00038088) was approved by Advarra Inc., the HEALing Communities Study Single Institutional Review Board (ClinicalTrials.gov Identifier: NCT04111939).

## Results

A total of 7,923 unique individuals received HCS KY TAP services through 30 partner agencies representing two sectors (healthcare and behavioral health) during the implementation period of January through December of 2023 across both programs, as shown in Table 1. Most individuals reached by these services were white (81.8%; 6,483), female (51.9%; 4,112) and between the ages of 35–54 years old (59.8%; 4,737). Over three-fourths (75.7%; 5,999) of these individuals resided in urban counties.

Costs for TAPs totaled $437,481.69 for all services provided over the course of 2023. The majority of transportation service costs were for fuel cards, Uber cards, and bus passes (40.3%; $176,310.50), followed by invoices for ride share, leased partner agency vehicle expenses, and transportation agencies (32.3%; $141,192.61) and costs for direct delivery services (27.4%, $119,978.58). The average cost per individual reached was $55.22 (Table 2).

The majority of individuals receiving transportation benefits (Urban = 5,999 vs. Rural = 1,924) and the majority of the money spent were in urban counties (Urban = 72.8%, $318,317.15 vs. Rural = 27.2%, $119,164.54). However, the average cost per individual reached was lower for those in urban counties ($53.06) compared to those in rural counties ($61.94), as shown in Table 2.

## Discussion

Considerable resources were invested in transportation services for HCS KY communities reflecting the true barrier this represents to many individuals seeking MOUD or other care. These direct investments in transportation were intended to improve retention in care for those receiving MOUD by preventing drop-out due to transportation barriers. Costs varied significantly by county type (urban/rural) with costs ranging from $27.52 (U5) to $109.93 (R3) per person reached. Results also identify wide variations within county types, which highlights the heterogeneity of need and available service options from one county to the next. The main type of transportation service provided also varied by county, with urban counties relying more on bus vouchers, fuel cards and ride-share services, while rural counties used more direct delivery approaches, suggesting the need to customize transportation programs to fit the local environment.

In terms of reach, urban counties reached many more individuals (5,999) than rural counties (1,924), which likely reflects variation in population size. Of the five urban counties, the largest by population reached 3,561 individuals. The volume and cost of TAP services provided varied widely across differing agencies across and within each county with many counties only having one or two agencies providing the bulk of services. Since the agency variability may have stemmed from agency resource limitations, and not community need, we did not summarize this as average cost per agency due to the inability to determine the unique agency differences, which may have led to their cost differences.

Although transportation challenges are often framed within the context of social determinants of health (SDOH), which include economic stability, access to education and healthcare, neighborhood and built environment, and social and community context, they represent just one piece of a larger set of obstacles to care (15, 29). Transportation needs overlap with several SDOH domains (e.g., access to care, infrastructure/built environment, social/community context) but addressing it effectively and sustainably requires a wider lens (11–14, 19).

Telehealth has emerged as an important modality for delivering all types of healthcare, including treatment for OUD, where research has already identified improvements in treatment access and retention (30, 31). Among these partner agencies, telehealth had greatly expanded during the COVID-19 pandemic, and in 2023, most still offered telehealth but also continued to provide in-person services, which is why lack of transportation was still considered a major barrier to care (19). As this trend of increased telehealth continues, it will be interesting to explore the impact it may have on the need for transportation support services.

Analysis of the HCS KY TAP survey implemented through the recovery coaching and care navigation programs identified several impediments to staying involved in treatment, with transportation being a primary obstacle. Common local barriers included lack of reliable vehicles (e.g., vehicle repair needs), limited public transportation, and resource limitations. Other barriers included the lack of economical ride service vendors, high fuel prices, and clients’ household disqualification from Medicaid transport. Medicaid limitations were especially important during the study period as vehicle ownership (even if the vehicle was inoperable) led to automatic disqualification for non-emergency medical transportation (NEMT) programs until July 2024 (19, 32). The available resources identified beyond those available for the clients themselves were limited to small grants and two vans operated by individual treatment centers. Even when public transportation was available within a county, clients may reside outside the available service area or experience scheduling challenges.

This study has some limitations. First, implementation of these programs required targeted staff resources, as each study county presented with differing obstacles. Agency partners encountered challenges with transportation assistance distribution, which may have limited the number of individuals who received services and the related expenses. Challenges included having sufficient resources required to identify transportation needs, build contractual agreements, and provide ongoing facilitation (e.g., meetings between implementation facilitators and agencies). These costs were not captured within this study and are therefore not reported but are real costs that would need to be considered before replicating this work. In addition, since agencies were required to submit demographic data on prior service use before receiving more transportation resources, those who did not provide data as required were unable to utilize the total funding made available for transportation support. Finally, staff turnover within each agency was often cited as a reason why services were not fully implemented or when data was lost or not collected. In situations where staff left their positions before a replacement was hired, there was no opportunity for cross-training or a hand-off of transportation support responsibilities. Therefore, data were limited by the availability of agency staff to collect and provide data to the research team. Turnover among recovery coaches and care navigators deployed in partner agencies also limited access to the recovery capital support provided. Since the transportation tracking process required a separate system for each agency, housed outside of their EMR, management of this program required staff time to document support and manually pull reports each month, which may have led to data retrieval errors. In follow-up meetings between the HCS KY team and partner agency staff, this turnover and added workload was mentioned as a reason transportation support was not being provided or data was missing.

## Conclusion

This analysis of the costs and reach of the HCS KY TAP supports the significance of transportation as a barrier to MOUD treatment, which is broadly experienced by individuals in both urban and rural settings. Transportation support is a critical area for investment to promote improved OUD treatment service delivery and to address SDOH in terms of access, infrastructure, and the environmental context among vulnerable population subgroups. Our findings demonstrate that targeted transportation support services are feasible and may effectively support the recovery journey of this vulnerable population. By building partnerships with local and regional service providers, programs can flexibly address treatment barriers and allocate resources to reach individuals seeking treatment. Research is needed to directly examine the associations between transportation and the likelihood of being retained on MOUD for at least six months, which has been proposed as the minimum effective treatment duration (33). Further study is also needed to determine which types of transportation assistance are most beneficial in rural areas and why average per person costs were so variable among counties. Additionally, policies that may allow reimbursements to healthcare providers delivering patient transportation services should be explored. Finally, the changes made to Medicaid transportation policies to clarify household working vehicle limitations which ensured all household members have transportation options were a valuable policy change for individuals needing these services.

## Figures and Tables

**Table 1.** Transportation Strategy Reach by County and Demographics

Individuals Reached		Rural			Urban					Total	%
		R1	R2	R3	U1	U2	U3	U4	U5		
	Total	1198	502	224	782	291	277	3561	1088	7923	
**Demographics**											
Sex											
	Male	486	254	115	378	134	126	1657	501	3651	46.1%
	Female	708	215	109	402	157	151	1793	577	4112	51.9%
	Other	2	1	0	1	0	0	7	0	11	0.1%
	No response	2	32	0	1	0	0	104	10	149	1.9%
Race/Ethnicity											0.0%
	Hispanic	2	2	3	5	3	0	37	6	58	0.7%
	Black	5	1	0	28	13	0	342	43	432	5.5%
	White	871	462	221	747	270	277	2664	971	6483	81.8%
	Non-Hispanic_Other	2	5	0	1	4	0	34	5	51	0.6%
	No response	318	32	0	1	1	0	484	63	899	11.3%
Age											0.0%
	<18	1	0	0	0	0	0	0	0	1	0.0%
	18–34	440	134	58	123	80	88	1016	243	2182	27.5%
	35–54	671	254	151	531	185	149	2051	745	4737	59.8%
	55+	86	80	15	127	26	39	390	92	855	10.8%
	No response	0	34	0	1	0	1	104	8	148	1.9%

**Table 2.** Transportation Strategy Cost by County and County Type

County Type		Vouchers*	Services**	Direct Delivery***	Total Cost Per County	Individuals Reached	Average Cost Per Individual Reached
**Urban**							
	U1	$ 32,640.00	$ 2,632.67	$ 20,379.23	$ 55,651.90	782	$ 71.17
	U2	$ 21,978.00	$ -	$ 2,641.97	$ 24,619.97	291	$ 84.60
	U3	$ 11,360.00	$ -	$ 510.51	$ 11,870.51	277	$ 42.85
	U4	$ 70,972.50	$ 93,045.78	$ 32,219.32	$ 196,237.60	3,561	$ 55.11
	U5	$ 6,600.00	$ 13,626.50	$ 9,710.67	$ 29,937.17	1088	$ 27.52
	**Urban Total**	$ 143,550.50	$ 109,304.95	$ 65,461.70	$ 318,317.15	5999	$ 53.06
**Rural**							
	R1	$ 11,860.00	$ 24,835.66	$ 26,518.78	$ 63,214.44	1,198	$ 52.77
	R2	$ 13,900.00	$ 7,052.00	$ 10,374.18	$ 31,326.18	502	$ 62.40
	R3	$ 7,000.00	$ -	$ 17,623.92	$ 24,623.92	224	$ 109.93
	**Rural Total**	$ 32,760.00	$ 31,887.66	$ 54,516.88	$ 119,164.54	1924	$ 61.94
**Overall Total**		$ 176,310.50	$ 141,192.61	$ 119,978.58	$ 437,481.69	7923	$ 55.22

‘-’ = no resources expended

* Vouchers = Uber cards, fuel cards and bus passes given to individuals identified by partner agencies

** Services = Ride share, vehicle expenses, and transportation services purchased by partner agencies and then invoiced to the University.

*** Direct delivery = Drivers’ time, mileage expenses, and other transportation services provided directly by Recovery Coaches

## Data Availability

Organization level data reported in the current study are not publicly available to protect the privacy of organizational partners.
